# Teleneuropsychology in Latin America: Experiences and challenges during the COVID-19 pandemic

**DOI:** 10.1590/1980-5764-DN-2025-0429

**Published:** 2026-03-06

**Authors:** Loreto Olavarria Vera, Claudia Dechent, Mario Parra Rodriguez, Lucía Crivelli, Nilton Santos Custodio Capuñay, Sonia Maria Dozzi Brucki, Maira Okada-Oliveira, Phillip Robert, Yakeel T. Quiroz, Andres Antivilo-Bruna, Alejandra Arboleda Ramirez, Patricia Lillo, María Agostina Carello, Teresa Torralva, Teresita Ramos, Claudia Duran-Aniotz, Ricardo Allegri, Andrea Slachevsky

**Affiliations:** 1 Geroscience Center for Brain Health and Metabolism, Santiago, Chile.; 2 Clínica Las Condes, Department of Neurology, Santiago, Chile.; 3 Universidad de Chile, Faculty of Medicine, Department of Psychiatry and Mental Health Oriente, Santiago, Chile.; 4 Universidad de Chile, Faculty of Medicine, Hospital Clínico Universidad de Chile, Department of Medicine, Santiago, Chile.; 5 University of Strathclyde, Department of Psychological Sciences & Health, Glasgow, Scotland.; 6 Fleni, Department of Cognitive Neurology, Buenos Aires, Argentina.; 7 Instituto Peruano de Neurociencias, Lima, Peru.; 8 Universidade de São Paulo, Faculdade de Medicina, Departamento de Neurologia, Grupo de Neurologia Cognitiva e do Comportamento, São Paulo SP, Brazil.; 9 Université Côte d’Azur CoBTeK lab, CreApolis JLNoisiez Fondation, Nice, France; 10 Massachusetts General Hospital, Harvard Medical School, Boston MA, United States.; 11 Universidad de Chile, Faculty of Medicine, Institute of Biomedical Sciences, Physiopathology Program, Neuroscience and East Neuroscience Department Neuropsychology and Clinical Neuroscience Laboratory, Santiago, Chile.; 12 Instituto Neurológico de Colombia, Medellín, Colombia.; 13 Institute of Cognitive Neurology, Buenos Aires, Argentina.; 14 Universidad de Chile, Faculty of Medicine and Hospital del Salvador, Memory and Neuropsychiatric Center, Santiago, Chile.; 15 Universidad Adolfo Ibanez, Latin American Brain Health Institute (BrainLat), Santiago, Chile; 16 Universidad de la Costa, Faculty of Health Sciences, Department of Neurosciences, Barranquilla, Colombia.; 17 Clínica Alemana-Universidad del Desarrollo, Neurology and Psychiatry Department, Santiago, Chile.

**Keywords:** Neuropsychology, COVID-19, Neuropsychological Tests, Latin America, Health Services Accessibility, Dementia, Neuropsicología, COVID-19, Pruebas Neuropsicológicas, América Latina, Accesibilidad a los Servicios de Salud, Demencia

## Abstract

The COVID-19 pandemic accelerated the use of teleneuropsychology (TeleNP) to deliver remote neuropsychological services in settings with limited clinic access.

**Objective:**

To examine TeleNP practices in Latin America (LA), focusing on clinicians’ perceptions of utility, validity, and barriers.

**Methods:**

A descriptive, exploratory cross-sectional survey was conducted between November 2020 and January 2021 among health professionals practicing neuropsychology in LA. The instrument, validated through a Delphi process, assessed professional background, TeleNP use, patient profiles, applied tests, and perceived advantages and challenges.

**Results:**

A total of 212 clinicians from 10 countries participated (mean age and clinical experience=42.7 years and 12.3 years, respectively). Participants were primarily psychologists (75.9%), but also neurologists, geriatricians, psychiatrists and speech-language pathologists. TeleNP adoption rose from 4.2% regular and 13.7% occasional pre-pandemic use to 58% at the time of the survey, with significant cross-country variation (χ^2^=79.0, df=30, p<0.001). TeleNP was used mainly for patient (90%) and informant (89.5%) interviews, screening (71.8%), and, in half of the cases, more extensive assessments. The advantages reported were improved access (81.5%), reduced transportation costs (79.8%), patient comfort (66.1%), and easier scheduling (66.1%). The main barrier identified was limited patient connectivity (84.7%). Regulatory knowledge was heterogeneous: 36.7% reported TeleNP authorization in their country, 23.5% reported no authorization, and 39.8% were unsure.

**Conclusion:**

TeleNP adoption in LA increased during the pandemic and is perceived as a valid, accessible modality to address geographic disparities in neuropsychological care. However, heterogeneous implementation, regulatory uncertainty, and technological limitations remain major challenges, underscoring the need for standardized guidelines.

## RESUMEN.

La pandemia de COVID-19 aceleró el uso de la teleneuropsicología (TeleNP) para ofrecer servicios neuropsicológicos remotos en entornos con acceso limitado a clínicas.

**Objetivo:**

Examinar las prácticas de TeleNP en América Latina (AL), centrándose en las percepciones de los clínicos sobre su utilidad, validez y barreras.

**Métodos:**

Se realizó una encuesta descriptiva, exploratoria y de corte transversal entre noviembre de 2020 y enero de2021 entre profesionales de la salud que ejercen la neuropsicología en AL. El instrumento, validado mediante un proceso Delphi, evaluó los antecedentesprofesionales, el uso de TeleNP, los perfiles de los pacientes, las pruebas aplicadas y las ventajas y desafíos percibidos.

**Resultados:**

Participaron un totalde 212 clínicos de 10 países (edad media=42,7 años; experiencia=12,3 años). Los participantes fueron principalmente psicólogos (75,9%), aunquetambién se incluyeron neurólogos, geriatras, psiquiatras y terapeutas del lenguaje. La adopción de TeleNP aumentó de un uso regular de 4,2% y un usoocasional de 13,7% antes de la pandemia a un 58% en el momento de la encuesta, con una variación significativa entre países (χ^2^=79,0; gl=30; p<0.001).La TeleNP se utilizó principalmente para entrevistas con pacientes (90%) e informantes (89,5%), cribado (screening) (71,8%) y, en la mitad de los casos,evaluaciones más exhaustivas. Las ventajas reportadas fueron la mejora de acceso (81,5%), la reducción de los costos de transporte (79,8%), la comodidaddel paciente (66,1%) y la facilidad de programación de citas (66,1%). La principal barrera identificada fue la limitada conectividad de los pacientes (84,7%).El conocimiento normativo fue heterogéneo: el 36,7% informó tener autorización para la TeleNP en su país, el 23,5% informó no tener autorización y el39,8% no estaba seguro.

**Conclusión:**

La adopción de TeleNP en AL aumentó durante la pandemia y se percibe como una modalidad válida y accesiblepara abordar las disparidades geográficas en la atención neuropsicológica. Sin embargo, la implementación heterogénea, la incertidumbre regulatoria y laslimitaciones tecnológicas siguen siendo desafíos importantes, lo que subraya la necesidad de directrices estandarizadas.

## INTRODUCTION

 Neuropsychological assessment is essential for diagnosing and managing brain disorders^1^ . Traditionally conducted in person using paper-and-pencil tasks, these evaluations were transformed by the COVID-19 pandemic, which accelerated the adoption of online methods collectively referred to as teleneuropsychology (TeleNP). Practiced for over two decades^2^ , TeleNP expanded markedly during the pandemic^3^ and improved access to services, particularly for historically underserved populations^4,5^ . 

 TeleNP — defined as the use of audiovisual tools for remote neuropsychological evaluation^6^ — offers advantages such as reduced travel time and lower costs^7^ , although it poses methodological challenges^8^ . Many clinicians still prefer in-person assessments, as traditional paper-and-pencil tests may not be fully transferable to remote formats^9,10^. 

 TeleNP introduces substantial changes in the clinical evaluation setting, modifying:

- clinician-patient interactions;
- the presentation of stimuli;
- the level of control clinicians maintains during assessments; and
- the ability to capture qualitative information from patient performance^11^

Another key factor is the technical infrastructure required, including stable internet and adequate electronic devices^6 ,12^. Access to these resources remains limited in Latin America due to disparities in connectivity and digital literacy, especially among older adults^13-15^. 

 A recent French pilot study examined the feasibility of TeleNP in older adults presenting with cognitive complaints^5^ . The study compared a basic neuropsychological assessment — including a clinical interview — administered via telemedicine with the same evaluation conducted face-to-face in a mobile clinic. Findings indicated no significant differences in acceptability or user experience between the two approaches, with participants reporting high levels of acceptance and user-friendliness for both the telemedicine format and the mobile clinic setting^4,5^. 

 In recent years, consensus-based guidelines have been published to establish standardized methodologies for TeleNP assessment^ 1,2,6 ,12,16^. However, information on TeleNP implementation in Latin America (LA) remains limited, particularly considering regional disparities in human and technological resources.^17,18^

 Considering these gaps and the growing reliance on TeleNP to reach underserved populations, this study sought to describe the use of TeleNP in LA during the COVID-19 pandemic and to examine clinicians’ perceptions of its utility, validity, and associated barriers. To address these objectives, a survey was conducted among neuropsychologists in the region. 

## METHODS

### Study design and subjects

 This study employed a descriptive, exploratory quantitative design. A cross-sectional online survey was conducted between November 2020 and January 2021 to collect data on the use of TeleNP across LA countries. 

 The survey targeted health professionals practicing neuropsychology with adult populations in LA. The sample was defined based on self-reported professional practice in neuropsychology, which was used as a proxy criterion given the heterogeneity of training pathways across the region and the lack of standardized certification in most countries^19^ . To maximize reach, the survey was distributed via email (Google Forms) through colleagues affiliated with the Latin America and the Caribbean Consortium on Dementia (LAC-CD) network^17^ , yielding a non-probability sample. 

 This study was approved by the Ethics Committee of the School of Medicine of Universidad de Chile (Approval Protocol No. 252-2020; Record No. 180). 

### Procedure

 The survey instrument was developed in Spanish, drawing on previously validated questionnaires to ensure content validity and methodological consistency with prior research^11,20,21^. To refine the instrument, a Delphi method was employed, involving iterative rounds of expert review to achieve consensus on item clarity, relevance, and comprehensiveness. The final survey was organized into five sections, each designed to capture distinct dimensions of TeleNP practice (Table 1). 

**Table 1:** Online survey details for Latin American neuropsychologists.

Section 1: Professional characteristics of participants.	The respondents’ professional background, training, and certifications in neuropsychology, and previous clinical experience were assessed.
Section 2: General antecedents of TeleNP use.	A general framework of teleneuropsychology use within the participant’s country and specific characteristics regarding the incorporation of technology into neuropsychological assessment were examined.
Section 3: Characterization of the population assessed via TeleNP.	Characteristics of patients evaluated using TeleNP, reasons for the assessment, and issues related to the clinical setting were documented.
Section 4: Characterization of clinical practice using TeleNP.	The main tests utilized and adaptations made for their application via TeleNP were indentified.
Section 5: Perception of the TeleNP use.	The perception of usefulness and validity of administering cognitive tests via TeleNP, as well as perceived barriers and challenges to implementing the method, were investigated.

Abbreviation: TeleNP: teleneuropsychology.

 The survey was translated into Portuguese by a Brazilian neuropsychologist (MO) and a neurologist (SB). Concordance between the Spanish and Portuguese versions was verified by a Chilean psychologist fluent in Portuguese. 

### Statistical analysis

 Survey data were analyzed using the Statistical Package for the Social Sciences (SPSS, version 27; SPSS Inc., Chicago, IL, USA). Descriptive statistics (frequencies, percentages, means, and standard deviations) were computed for each item to characterize the sample and overall patterns of TeleNP use. To examine associations between categorical variables, chi-square tests of independence were conducted, including analyses of cross-country differences in TeleNP practices and reported regulations, as well as the relationship between training background and TeleNP use. Statistical significance was set at p<0.05. 

## RESULTS

### Sociodemographic profile and professional background of study participants

 A total of 212 neuropsychologists from 10 LA countries responded to the survey. The mean age of participants was 42.72 (standard deviation – SD=10.6), with an average of 12.28 years of professional experience (SD=8.7). Most participants were psychologists (75.9%), although there were also neurologists (9%), geriatricians (5.2%), speech therapists (4.7%), and psychiatrists (1.4%). Table 2 for country-level details. 

**Table 2:** Sociodemographics and Professional background of study participants by country (n=210).

		Argentina	Brazil	Chile	Colombia	Ecuador	Mexico	Peru	Total
**Sociodemographic characteristics (%)**										
	Study participants, n (%)	21 (9.4)	97 (45.8)	26 (12.3)	23 (10.8)	12 (5.6)	11 (5.1)	20 (9.4)	210 (100)
	Age, mean (SD)	40.48 (12.8)	44 (10.2)	42.62 (9.27)	37.55 (9.62)	42.92 (10.7)	39.1 (11.3)	47.68 (11.3)	42.75 (10.6)
	Years of experience, mean (SD)	11.9 (9.1)	12.34 (8.94)	13.96 (8.04)	10.52 (8.1)	10.92 (7.4)	9.64 (9.78)	15.42 (9.3)	12.29 (8.7)
**Professional background (%)**										
	Psychologist, n (%)	16 (76.2)	62 (64.6)	22 (84.6)	22 (95.7)	7 (58.3)	11 (100)	18 (94.7)	160 (75.5)
	Neurologist, n (%)	0 (0)	14 (14.6)	2 (7.7)	0 (0)	2 (16.7)	0 (0)	1 (5.3)	19 (9)
	Geriatricians, n (%)	1 (4.8)	10 (10.4)	0 (0)	0 (0)	0 (0)	0 (0)	0 (0)	11 (5.2)
	Speech therapist, n (%)	3 (14.3)	4 (4.2)	1 (3.8)	1 (4.3)	1 (8.3)	0 (0)	0 (0)	10 (4.7)
	Psychiatrists, n (%)	1 (4.8)	1 (1)	0 (0)	0 (0)	1 (8.3)	0 (0)	0 (0)	3 (1.4)
	Other professional background, n (%)	0 (0)	0 (0)	1 (3.8)	0 (0)	1 (8.3)	0 (0)	0 (0)	4 (1.9)

**Figure 1:**
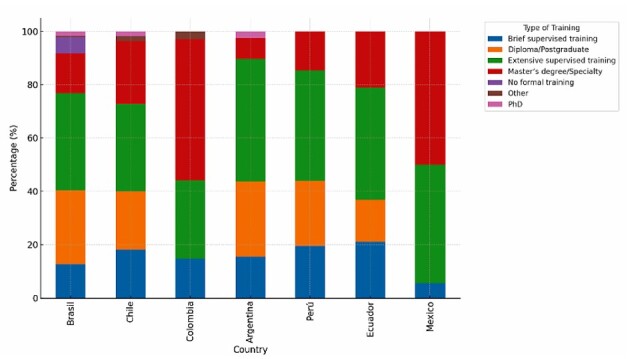
Neuropsychology Training by Country in Latin America and the Caribbean.

 The educational background and type of training in neuropsychology varied among respondents: 22.2% reported holding a diploma or postgraduate certificate, 20.7% a master’s degree or specialty in the field, and 1.3% a PhD. In terms of practical training, 37.5% had received extensive supervised training, whereas 14.7% reported only brief supervised experience in neuropsychology (Figure 1 for country-level details). 

 Regarding time allocation for neuropsychological evaluations within the working day, 40.1% reported it as their primary work activity; 39.6% dedicated only part of their working day, and 20.3% considered it a minor activity. Most respondents reported the private sector as their primary workplace (61.5%), followed by the public sector (32.0%) and academia (4–5%). 

### General antecedents of the use of teleneuropsychology

 Overall, 125 participants (58%) reported currently practicing TeleNP, representing an increase compared with the period before the COVID-19 pandemic. Prior to the pandemic, its use was infrequent: 4.2% reported using it regularly and 13.7% occasionally. 

**Figure 2:**
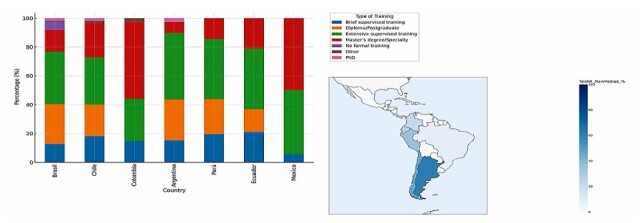
Use of teleneuropsychology in Latin America and the Caribbean per country and Geographical Distribution of Teleneuropsychology as Main Evaluation Method in Latin America.

 At the time of the survey, 22.6% of respondents reported using TeleNP as their primary evaluation method. A mixed approach — combining remote and in-person assessments — was employed by 36.8%. Meanwhile, 33.5% relied exclusively on in-person evaluations, and 7.1% had suspended their clinical practice altogether. The analysis revealed statistically significant cross-country differences in the current practice of TeleNP (χ^2^=79.0, df=30, p<0.001). In Argentina and Chile, a large proportion of professionals reported using TeleNP as their primary evaluation method, whereas in Brazil fully in-person assessments predominated. In contrast, Colombia and Peru showed greater reliance on mixed approaches, with Peru also presenting the highest proportion of professionals who reported suspending their clinical practice. Figure 2 shows the practice of TeleNP by country. 

 When exploring the association between training background and the use of TeleNP, the analysis did not reveal significant differences across groups. Professionals with postgraduate diplomas, master’s or specialty training, doctoral degrees, or varying levels of supervised practice were similarly distributed among those who reported using TeleNP as their main method, those combining it with in-person practice, and those maintaining fully in-person evaluations. The chi-square test confirmed the absence of a statistically significant relationship (χ^2^=19.23, df=18, p=0.378), suggesting that the type of academic or supervised training does not appear to be a decisive factor in shaping current patterns of TeleNP use across the region. 

 Regarding technology use in TeleNP, most participants preferred the Zoom platform (52.8%) and the use of a computer or laptop (80.2%). The latter was particularly prevalent in Chile and Argentina, where participants from Peru, Brazil, Colombia, and Mexico preferred cell phone for TeleNP. The predominant type of internet connection was Wi-Fi (90.6%). 

 The analysis of national regulations on TeleNP revealed substantial cross-country variation. Overall, 36.7% (n=76) of respondents reported that TeleNP is authorized in their country, 23.5% (n=49) indicated it is not, and 39.8% (n=82) were unsure (χ^2^=87.90, df=12, p<0.001). In Argentina, nearly all affirmed authorization (95%, n=20), while uncertainty predominated in Chile (≈60%, n=15) and Brazil (44%, n=45). Most respondents in Colombia reported authorization (74%, n=17), whereas in Peru the majority reported no authorization (68%, n=13). In Ecuador and Mexico, "don’t know" was the most frequent response. These findings highlight both regulatory diversity and limited awareness of the legal status of TeleNP in the region. 

 TeleNP was mainly used with patients showing mild cognitive impairment or subjective complaints (42.9%) (Table 3 for country-level details). 

**Table 3:** Clinical characteristics of patients assessed using TeleNP by country (n=210).

Characteristic		Countries							
		Argentina (n=21)	Brazil (n=96)	Chile (n=26)	Colombia (n=23)	Ecuador (n=12)	Mexico (n=11)	Peru (n=20)	Total (n=210)
Type of population, n (%)									
	Pediatric	0 (0)	6 (6.3)	2 (7.7)	8 (34.8)	6 (50)	0 (0)	7 (36.8)	30 (14.2)
	Adult	17 (81)	11 (11.5)	13 (50)	14 (60.9)	5 (41.7)	4 (36.4)	11 (57.9)	77 (36.3)
	Older adult	17 (81)	26 (27.1)	13 (50)	8 (34.8)	5 (41.7)	4 (36.4)	6 (31.6)	80 (37.7)
Clinical condition, n (%)									
	MCI	18 (85.7)	29 (30)	14 (53.8)	13 (56.5)	6 (50)	2 (18.2)	7 (36.8)	91 (42.9)
	Dementia	11 (52.4)	19 (19.8)	9 (34.6)	8 (34.8)	3 (25)	3 (27.3)	3 (15.8)	58 (27.4)
	Stroke	13 (61.9)	10 (10.4)	7 (27)	7 (30.4)	2 (16.7)	0 (0)	2 (10.5)	42 (19.8)
	ADDi	12 (57.1)	19 (19,8)	9 (34.6)	13 (56.5)	4 (33.3)	0 (0)	11 (57.9)	69 (32.5)
	TBI	4 (19)	8 (8,3)	6 (23.1)	8 (34.8)	1 (8.3)	0 (0)	1 (5.3)	30 (14.2)
	Epilepsy	2 (9.5)	3 (3.1)	3 (11.5)	1 (4.3)	4 (33.3)	0 (0)	2 (10.5)	16 (7.5)
	SCC	17 (81)	31 (32,3)	14 (53.8)	12 (52.2)	4 (33.3)	5 (45.5)	7 (36.8)	91 (42.9)
	Multiple sclerosis	1 (4.8)	1 (1)	1 (3.8)	0 (0)	0 (0)	0 (0)	0 (0)	5 (2.4)
	Psychiatric disorder	5 (23.8)	15 (15.6)	5 (19.2)	4 (17.4)	4 (33.3)	4 (36.4)	6 (31.6)	44 (20.8)

Abbreviations: MCI, Mild cognitive impairment; ADD, Attention deficit disorder; TBI, Traumatic brain injury; SCC, Subjective cognitive complaints.

### Characterization of clinical practice using Teleneuropsychology

 Regarding the purpose of TeleNP, most respondents reported using it to interview both patients (n=112, 90%) and family members or informants (n=111, 89.5%). TeleNP was also frequently applied for screening evaluations (n=89, 71.8%), whereas only half of the respondents (n=62, 50%) reported using it to conduct extensive assessments. 

**Figure 3:**
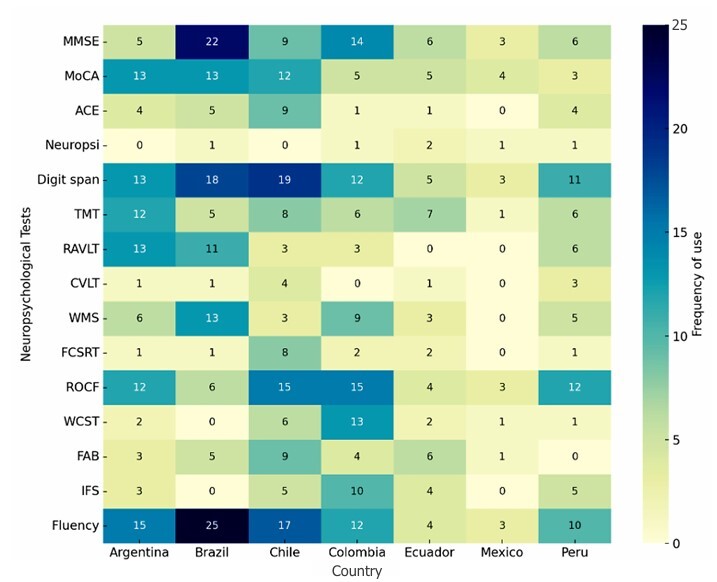
Neuropsychological Test used in teleneuropsychology by Country. Abbreviations: MMSE, Mini-Mental State Examination; MoCA, Montreal Cognitive Assessment; ACE, Addenbrooke’s Cognitive Examination; TMT, Trail Making Test; RAVLT, Rey Auditory Verbal Learning Test; CVLT, California Verbal Learning Test; WMS, Wechsler Memory Scale; FCSRT, Free and Cued Selective Reminding Test; ROCF, Rey-Osterrieth Complex Figure; WCST, Wisconsin Card Sorting Test; FAB, Frontal Assessment Battery; IFS, INECO Frontal Screening.

 The most frequently used test in TeleNP was Verbal Fluency (70.2%), followed by the Digits Span task (66.1%), the Rey-Osterrieth Complex Figure Test (54.8%), and the Mini-Mental State Examination (53,2%). Figure 3 displays the distribution of neuropsychological tests administered through TeleNP across LA countries. 

 A total of 60.5% of respondents reported adapting test administration protocols compared with traditional in-person assessments. Most of these adaptations were self-devised (42.7%), while others followed modifications proposed by test authors (22.6%) or expert consensus (18.5%). Reported strategies included incorporating an informant to assist with the presentation of test stimuli, sending stimuli in advance by email, or displaying them through screen-sharing slides. 

### Perception of teleneuropsychology

 Overall, 80.6% (n=100) of the participants considered that TeleNP was sufficient to fulfill the goal of a neuropsychological evaluation. Reported advantages of TeleNP included improved access regardless of location (81.5%); reduced transportation costs (79.8%); greater patient comfort from being assessed in a familiar environment (66.1%); and easier scheduling (66.1%). Additional benefits mentioned by participants included lower exposure to COVID-19 infection in an at-risk population and savings in travel time. Detailed country-level results are presented in Table 4. 

**Table 4:** Advantages of Teleneuropsychology per country (n=124).

Advantages of TeleNP	Argentina (n=16)	Brazil (n=41)	Chile (n=21)	Colombia (n=16)	Ecuador (n=9)	Mexico (n=6)	Peru (n=14)	Total (n=124)
Accessibility (%)	16 (100)	16 (39)	20 (95.2)	15 (93.8)	8 (88.9)	5 (83.3)	11 (78.6)	101 (81.5)
Decrease in transportation costs (%)	14 (87.5)	26 (63.4)	19 (90.5)	14 (87.5)	8 (88.9)	6 (100)	11 (78.6)	99 (79.8)
Appreciation of Patient satisfaction (%)	5 (31.3)	9 (22)	5 (23.8)	8 (50)	2 (22.2)	6 (100)	4 (28.6)	34 (27.4)
Ease of appointment scheduling (%)	10 (62.5)	22 (53.7)	16 (76.2)	10 (62.5)	4 (44.4)	3 (50)	9 (64.3)	74 (59.7)
Patient comfort in the home environment (%)	12 (75)	24 (58.5)	18 (85.7)	9 (56.3)	6 (66.7)	3 (50)	9 (64.3)	82 (66.1)

Abbreviation: TeleNP: teleneuropsychology.

 Patient satisfaction was reported by 27.4% (n=34) of participants in the total sample, with important variation across countries. Rates ranged from 100% (n=6) in Mexico to 22% (n=9) in Brazil. In Chile, the proportion was 23.8% (n=5), in Argentina 31.3% (n=5), in Peru 28.6% (n=4), in Colombia 50% (n=8), and in Ecuador 22.2% (n=2). 

 The main barrier reported by participants to TeleNP across most countries was the limited connectivity of the evaluated individuals, which was poorer than that of the evaluator (additional details in Table 5). Indeed, restricted access to technology and inadequate connectivity in LA were identified as the principal barriers to the widespread implementation of TeleNP in the region. 

**Table 5:** Technical Difficulties and main challenges of Teleneuropsychology (n=124).

Difficulties in TeleNP		Argentina (n=16)	Brazil (n=41)	Chile (n=21)	Colombia (n=16)	Ecuador (n=9)	Mexico (n=6)	Peru (n=14)	Total (n=124)
Technical issues. n (%)									
	Connectivity	16 (100)	28 (68)	18 (86)	16 (100)	9 (100)	3 (50)	14 (100)	105 (84.7)
	Evaluator connectivity	6 (37.5)	7 (17)	8 (38.1)	6 (37.5)	3 (33.3)	3 (50)	4 (29)	37 (29.8)
	Sound quality	5 (31.3)	10 (24)	5 (23.8)	10 (62.5)	2 (22.2)	3 (50)	5 (35.7)	40 (32.3)
	Video quality	2 (12.5)	11 (27)	8 (38.1)	8 (50)	6 (66.7)	3 (50)	4 (29)	42 (33.9)
Difficulties during the sessions due to clinical issues (%)									
	Sensory problems	9 (56.3)	23 (51)	14 (66.7)	10 (62.5)	7 (77.8)	3 (50)	8 (57.1)	74 (59.7)
	Interruption	7 (43.8)	17 (41)	8 (38.1)	12 (75)	5 (55.6)	2 (33.3)	9 (64.3)	60 (48.4)
	Trouble understanding instructions	4 (25)	10 (24)	10 (47.3)	10 (62.5)	5 (55.6)	4 (66.7)	5 (37.7)	48 (38.7)
	Little familiarity with technology	6 (37.5)	10 (24)	11 (52.4)	8 (50)	5 (55.6)	3 (50)	6 (43)	49 (39.5)
	No issues reported	1 (6.3)	2 (4.9)	1 (4.8)	0 (0)	0 (0)	0 (0)	0 (0)	5 (4)

## DISCUSSION

 This study provides one of the first region-wide overviews of TeleNP practices in LA during the COVID-19 pandemic. Findings indicate that although TeleNP was rarely used before the pandemic, its adoption increased substantially, with more than half of professionals reporting its use. The application of TeleNP varied significantly across countries: in Argentina and Chile, many clinicians reported using it as their primary evaluation method; in Brazil, fully in-person assessments predominated; and in Colombia and Peru, mixed approaches were more common. Clinically, TeleNP was most frequently applied to patients with mild cognitive impairment and subjective cognitive complaints and was primarily used for interviews and screening evaluations rather than full assessments. Despite notable barriers — particularly technological limitations — most clinicians perceived TeleNP as useful and advantageous, emphasizing accessibility, reduced costs, and greater patient comfort as its main benefits. 

 Consistent with these results, a recent national survey in Mexico^22^ similarly showed a significant rise in TeleNP implementation after the onset of the pandemic, with more than 80% of neuropsychologists reporting current use. Their data also revealed that training in TeleNP was predominantly acquired through self-directed learning and bibliographic review rather than formal supervised instruction, and that technological barriers — such as poor internet connectivity and limited access to adequate devices — were among the most frequently reported obstacles. Serrano-Juárez et al.^23^ emphasized that this rapid and largely unstructured expansion of TeleNP in the region should be systematically organized through models tailored to Latin American contexts, including standardized procedures, technological requirements, and culturally sensitive guidelines to ensure validity, reliability, and ethical soundness in clinical practice. 

 TeleNP is an emerging field that has expanded in recent years, particularly accelerated by the COVID-19 pandemic, and its use continues to be reported in current practice^24^. Although general practice recommendations have been recently developed to support TeleNP implementation^6,12^, no studies had previously examined its application in the context of the pandemic across LA, a region characterized by significant cultural diversity and marked heterogeneity in technological infrastructure. Importantly, while participants recognized multiple advantages of TeleNP, they also highlighted persistent challenges in LA, particularly limited access to adequate technology and internet connectivity, as well as the need to adapt traditional in-person assessment procedures to remote modalities. 

 The findings highlight patterns that mirror, but also diverge from, those reported in other regions. Similar to surveys conducted in North America and Europe, the use of TeleNP in LA was rare before COVID-19 but increased substantially during the pandemic, particularly for patient and informant interviews and brief cognitive screening^25,26^. However, in contrast to high-income countries where access to adequate devices and stable internet is more widely available, clinicians in LA consistently identified patient-side connectivity as the main barrier, echoing concerns raised by previous studies in underserved contexts^5,27^. The heterogeneity across countries in legal frameworks and clinicians’ awareness of them also appears more pronounced than in settings with centralized telehealth regulations^28^. Taken together, these results suggest that while the advantages of TeleNP — such as accessibility and reduced costs — are broadly recognized worldwide, the LA experience underscores how structural inequalities in infrastructure and digital literacy critically shape the feasibility and equity of remote neuropsychological assessment. 

 Regarding technological resources, most participants reported conducting assessments using a computer or laptop, while only a small group relied on cell phones. 

 This finding is consistent with reports from other regions^25^, where TeleNP has also been primarily implemented via computers. Such preference is likely shaped by international recommendations on screen size for stimulus presentation^6^ and by the fact that most psychometric validation studies of TeleNP have been carried out on laptops or computers^8^. Nevertheless, this poses a significant challenge in LA, where large segments of the population — particularly underserved groups — have more reliable access to cell phones than to computers. Ensuring equitable implementation of TeleNP will therefore require strategies to improve access to adequate devices^5^. Additional barriers included unstable internet connections, often aggravated during the pandemic by increased network demand due to teleworking and online education^29^. This limitation is particularly concerning in rural areas, where connectivity is typically poorer than in urban centers, even though TeleNP could be especially beneficial for these populations^30-32^. 

 In terms of regulation, only one third of participants indicated that TeleNP was legally authorized in their country, while nearly 40% were uncertain. This suggests that, beyond the heterogeneity in local regulatory frameworks, there is limited awareness among professionals about the legal status of TeleNP in their own contexts. This gap highlights the need for clearer guidance and dissemination of information for clinicians, similar to the standards provided by the American Psychological Association^28^. Notably, since December 2022, Brazil has implemented a telehealth law establishing regulatory provisions for both patients and health professionals, illustrating how country-level developments may not always be fully known or understood by practitioners. 

 Despite its limitations, clinicians in LA viewed TeleNP as valid, accessible, and patient-centered. These included reduced transportation costs and the comfort of being assessed at home, given that traveling to a medical facility can be stressful in itself^4^, particularly for the populations most frequently attended by the professionals surveyed. TeleNP was also recognized as a valuable option for individuals with physical disabilities, enabling them to receive quality care without leaving their homes^25^. Similarly, for patients living in remote rural areas, TeleNP offers the possibility of avoiding long and costly trips to access specialized healthcare^33^. From an economic standpoint, telemedicine has further been shown to reduce healthcare costs and require less time compared with traditional in-person approaches^34^. 

### Challenges encountered by professionals

 Adapting neuropsychological assessments to TeleNP in LA proved challenging, as clinicians were required to introduce multiple modifications to ensure effective evaluations. Among the most common adjustments were changes to test instructions, alternative modes of stimulus presentation, and, in some cases, the involvement of a family member to assist the patient — an approach rarely used in traditional face-to-face settings. These modifications also generated additional difficulties, particularly the limited control over the evaluation environment, which demanded greater vigilance from examiners to monitor patient behavior and manage potential distractions. Clinicians often faced the delicate task of balancing the supportive role of an informant with the risk of excessive interference in the assessment process, an issue identified as a specific challenge for TeleNP practice. 

 A key limitation of this study concerns the potential selection bias associated with the recruitment strategy. The survey was primarily disseminated through the LACCD network, which may have favored the participation of professionals from countries with stronger digital connectivity and international academic ties. Consequently, contexts with more consolidated infrastructure and professional networks may be overrepresented, whereas those with fewer technological resources or reduced integration into regional collaborations may be underrepresented. This limitation restricts the generalizability of the findings and underscores the need for future studies to adopt more inclusive sampling strategies to better represent the diversity of regional realities. 

 In conclusion, this study provides essential insights into the implementation of TeleNP across Latin America, highlighting both its rapid expansion during the COVID-19 pandemic and the persistent challenges that remain. While TeleNP has demonstrated its potential to improve access to neuropsychological services, particularly for underserved populations, its equitable use is still constrained by technological barriers and disparities in digital literacy. Moreover, psychometric validation of remote assessments in diverse populations remains an urgent priority to ensure methodological rigor. Moving forward, the systematic development of regional guidelines — including standardized procedures, platform and device requirements, and context-sensitive recommendations — will be critical to consolidate TeleNP as a reliable, valid, and sustainable practice beyond the pandemic. 

## Data Availability

The datasets generated and/or analyzed during the current study are available from the corresponding author upon reasonable request.
